# Supplementary material for: All-at-once RNA folding with 3D motif prediction framed by evolutionary information
Source: Nat Methods. 2025 Oct 3;22(10):2094–106. doi: 10.1038/s41592-025-02833-w (PMC12510878; doi:10.1038/s41592-025-02833-w)
Supplement: Supplementary file 3 — Source code to run CaCoFold-R3D. Data and results. [file 41592_2025_2833_MOESM3_ESM.gz › supplemental_material/results/CaCoFold-R3D/CaCoFold-R3D_Rfam/RF01935_bxd_7.R2R.sto.pdf]

RF01935_bxd_7

5′ - U G U A U U U U U A G G U A U C C G U A U A A U U G A C U U U U U G A C U U U G R A Y Y G C ● Y ● G U ● G C A G G U C G U U G A C U
